# An inhibitor of K^+ ^channels modulates human endometrial tumor-initiating cells

**DOI:** 10.1186/1475-2867-11-25

**Published:** 2011-08-02

**Authors:** Brandon M Schickling, Nukhet Aykin-Burns, Kimberly K Leslie, Douglas R Spitz, Victoria P Korovkina

**Affiliations:** 1 Department of Obstetrics and Gynecology, University of Iowa, 200 Hawkins Drive, Iowa City, IA 52242, USA; 2 Department of Radiation Oncology, University of Iowa, 200 Hawkins Drive, Iowa City, IA 52242, USA; 3 Free Radical and Radiation Biology Program, University of Iowa, B180 Med Labs, Iowa City, IA 52242, USA; 4 Holden Comprehensive Cancer Center, University of Iowa, 200 Hawkins Drive, Iowa City, IA, 52242, USA

**Keywords:** endometrial cancer, potassium channels, cancer stem cells, tumor initiating cells

## Abstract

**Background:**

Many potassium ion (K^+^) channels function as oncogenes to sustain growth of solid tumors, but their role in cancer progression is not well understood. Emerging evidence suggests that the early progenitor cancer cell subpopulation, termed tumor initiating cells (TIC), are critical to cancer progression.

**Results:**

A non-selective antagonist of multiple types of K^+ ^channels, tetraethylammonium (TEA), was found to suppress colony formation in endometrial cancer cells via inhibition of putative TIC. The data also indicated that withdrawal of TEA results in a significant enhancement of tumorigenesis. When the TIC-enriched subpopulation was isolated from the endometrial cancer cells, TEA was also found to inhibit growth in vitro.

**Conclusions:**

These studies suggest that the activity of potassium channels significantly contributes to the progression of endometrial tumors, and the antagonists of potassium channels are candidate anti-cancer drugs to specifically target tumor initiating cells in endometrial cancer therapy.

## Introduction

Potassium (K^+^) ion channels are important contributors to the malignant phenotype in cancer cells and as such have been shown to drive progression of cancers of the breast, prostate, endometrium and brain [1-8]. Multiple mechanisms exist by which K^+ ^channels exert their oncogenic functions. For example, K^+ ^channels have been shown to modulate cell cycle progression to increase cell proliferation as well as promote cytoskeletal remodeling to enhance invasion and migration [9-21]. Inhibitors of K^+ ^channels thus constitute putative anti-cancer drugs [1,2,22-26], though to date none of these antagonists have been explored in a clinical trial setting for any type of cancer.

Novel developments in cancer research demonstrate that tumor initiating cells (TIC, also referred to as cancer stem cells) cause the onset and recurrence of cancers [27-29]. Several biological agents that aim to eradicate TIC are currently in phase I/II clinical trials, but a clinical need remains to identify other pharmacologic approaches to prevent TIC-mediated tumorigenesis.

Interestingly, K^+ ^channels genes have been shown to be amplified in cancers, but the roles of K^+ ^channels in TIC and by extension in cancer progression have not been rigorously addressed. In this manuscript, we present novel observations that an inhibitor of multiple types of K^+ ^channels, tetraethylammonium (TEA), abrogates the tumorigenic abilities of a TIC-enriched subpopulation derived from human endometrial cancer cells and thus may represent a therapeutic strategy for endometrial cancer therapy.

## Results and Discussion

Earlier studies have suggested that, in certain cancers, K^+ ^channels accelerate tumor growth; however, their role in the cancer progression remains unclear [1]. Herein, we examined the importance of the integrated activity of multiple K^+ ^channels in the establishment of new endometrial tumors and their putative roles in the onset or recurrence of the disease. Cells used in these studies model two clinically relevant types of endometrial cancers: 1) Ishikawa H cells represent a hormone-dependent type (i.e., ER- and PR-positive); and 2) Hec50co cells are a model of a hormone-independent type (i.e., ER and PR-negative) [30]. K^+ ^channels as diverse as voltage-gated (e.g., HERG) and calcium-sensitive (e.g., IKCa) have been reported to regulate the progression of endometrial cancers [31,32]. Therefore, we utilized tetraethylammonium (TEA), a broad inhibitor of many types of K^+ ^channels, instead of siRNA-mediated silencing of individual K^+ ^channels to examine the general integrated role of voltage-gated, calcium-sensitive, and ATP-sensitive K^+ ^channels in tumorigenesis.

**Figure 1:**
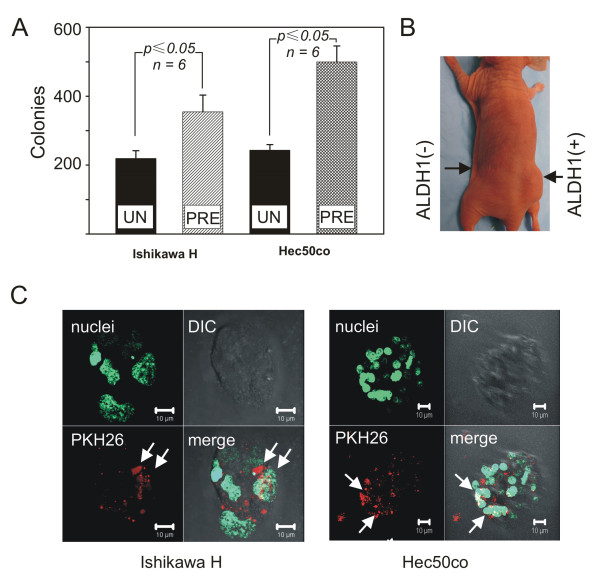
**Presence of TIC subpopulation in endometrial cancer cells**. (A) Ishikawa H cells untreated (*Ishikawa H, solid bar*) or pre-treated with TEA (*Ishikawa H, shaded bar*) were seeded onto TEA-free soft agar and cell colonies were counted 21 days later. Similarly, Hec50co cells untreated (*Hec50co, solid bar*) or pre-incubated with TEA (*Hec50co, shaded bar*) were grown in TEA-free agar for 21 days. p ≤ 0.05 vs. untreated for respective cell line; N = 3. (B) Hec50co cells were separated into the sub-populations with high (*ALDH1 (+)*) and low (*ALDH1 (-)*) activity of the enzyme ALDH1 via fluorescent cell sorting. Athymic mice were injected with 100,000 ALDH1(+) or ALDH1(-) cells and inspected for tumor growth. N = 2. (C) Ishikawa H endospheres (*left panel,*) and Hec50co endospheres (*right panel*) were cultured in the presence of a cell membrane marker PKH26 (*red*) and counter-stained with the live cell DNA marker CYTO16 (*green*). Visualization and a three dimensional reconstruction of images were performed using a Zeiss LSM 510 confocal imaging system. N = 5 (Ishikawa H) and N = 8 (Hec50co).

Earlier reports have documented the growth-promoting effects of K^+ ^channels in tumors of various origins [4,14,15]. We therefore hypothesized that cells pre-treated with an inhibitor of K^+ ^channels will form fewer colonies in soft agar. Since most anti-cancer therapies are acute and not chronic treatment regimens, we first examined how transient exposure to TEA alters the subsequent ability of endometrial cancer cells to form new colonies in the absence of TEA. Endometrial cancer cells pre-treated with TEA for 48 h had no difference in viability as compared to untreated cells as determined by trypan blue exclusion (data not shown). Next, endometrial cancer cells were pre-treated with TEA, seeded onto TEA-free soft agar, and cell colonies visualized by crystal violet staining three weeks after seeding (Figure 1A). Unexpectedly, Ishikawa H cells pre-treated with TEA (Figure 1A, *Ishikawa H cells, PRE*, shaded bar) exhibited an increased efficiency in formation of new colonies when compared to the untreated controls (Figure 1A, *Ishikawa H cells, UN*, solid bar). Similarly, treated Hec50co cells (Figure 1A, *Hec50co cells, PRE*, shaded bar) showed augmented numbers of colonies in contrast to the untreated cells (Figure 1A, *Ishikawa H cells, UN*, solid bar). These findings indicate that an enhancement of a tumorigenic ability occurs following a transient exposure and/or subsequent withdrawal of TEA.

Since newly arising primary and metastatic tumors are thought to reflect the presence of TIC [28,29,33], we hypothesized that the observed potentiation of tumorigenicity is due to activation of TIC in our cell models. Therefore, we isolated TIC-enriched subsets from the Ishikawa H and Hec50co cells to examine whether TEA regulates these sub-populations. Identification of the TIC subpopulation from other cancer cell types is challenging due to their rarity and uncertainties as to TIC-specific markers. It has been suggested that one difference between TIC and other cancer cells is a lower rate of cell division and an elevated activity of the enzyme aldehyde dehydrogenase isoform 1 (ALDH1) [28,29,33-35]. We utilized both of these properties to identify and isolate the TIC-enriched subpopulation from Ishikawa H and Hec50co endometrial cancer cells. First, we isolated the cells with high ALDH1 activity, *ALDH1 (+)*, and compared their tumorigenic capacity to those with low ALDH1 activity, *ALDH1 (-)*, using mouse xenograft models. Cells with either high or low ALDH1 activity, as determined by ALDEFLUOR assays, were injected into the flanks of nude mice and tumor growth measured 45-60 days later. Figure 1B shows a tumor in the flank of a mouse where 100,000 ALDH1 positive Hec50co cells have been injected (Figure 1B, *ALDH1 (+)*). In contrast, no tumor formed in the opposite flank that was injected with 100,000 cells with low ALDH1 activity (Figure 1B, *ALDH1 (-)*). We thus concluded that a population of TIC is present in our cell models and they can be identified by the high activity of ALDH1 enzyme.

It has been suggested that not only TIC themselves but their early progeny also can demonstrate high ALDH1 activity. As a consequence, ALDH1 positive cells contain two sub-populations: (1) the rapidly proliferating early progenitor cells; and (2) TIC with a stem cell-like low proliferation rate or quiescent TIC. Our subsequent approaches therefore were to differentiate TIC in our cell models on the basis of their low proliferative rate in addition to the high ALDH1 activity. Specifically, we visualized potential TIC using a fluorescent cell membrane marker, PKH26, that is incorporated into the living cells. Retention of PKH26 dye in cell membranes is a function of cell division, whereby the more times cells divide, the less dye each cell retains [33,36]. For these studies, we used a modified clonogenic assay (endosphere culture, see Methods), and we expected that we could use PKH26 fluorescence intensity to differentiate between the proliferating progenitor cells and the slowly dividing or quiescent TIC in endospheres. Here we present images of live Ishikawa H and Hec50co endospheres. The left panel in Figure 1C is a 3-dimensional reconstruction of an Ishikawa H endosphere (Figure 1C, *left panel*, nuclei, green) where only a few cells, presumed to be TIC (indicated by the white arrows), show high retention of a PKH26 dye (Figure 1C, *left panel*, PKH26, red). This endosphere was additionally visualized using a transmitted light (Figure 1C, *left panel*, DIC, grey) and subsequently all images were merged (Figure 1C, *left panel*, merge, white arrows point at putative TIC). In Hec50co endospheres, (Figure 1C, *right panel*, nuclei, green) we observed similar distribution of PKH26 dye (Figure 1C, *right panel*, PKH26, red) where white arrows indicate putative TIC. The fluorescent and transmitted light (Figure 1C, *right panel*, DIC, grey) images were merged (Figure 1C, *left panel*, merge, white arrows designate TIC). We thus concluded that putative TIC are present in our cell models and can be identified by the high activity of ALDH1 enzyme. Importantly, these cells may be the originators of new tumor spheres.

**Figure 2:**
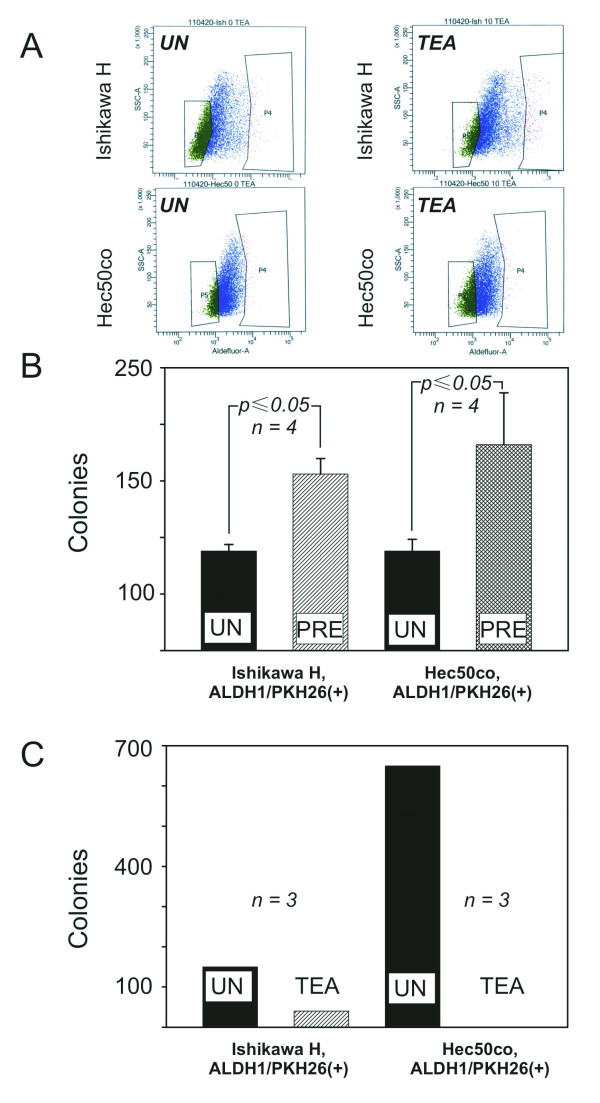
**Effect of TEA on tumorigenic potential of TIC**. (A) Ishikawa H (upper panels) or Hec50co (lower panels) cells were propagated in the presence of a cell membrane marker PKH26 and either kept untreated (left panels) or treated with TEA (right panels). PKH26(+) cells were isolated, and then ALDH1(+) cells obtained from that population by fluorescent cell sorting. Cells in the box in the right of the plots were collected for the soft agar assay in Figure 2B. N = 3. (B) Ishikawa H cells untreated (*Ishikawa H ALDH1/PKH26(+)*, UN, *solid bar*) or pre-treated with TEA (*Ishikawa H ALDH1/PKH26(+)*, PRE, *shaded bar, p ≤ 0.05*) were separated by the fluorescent cell sorting. ALDH1/PKH26 positive fractions were seeded onto the TEA-free soft agar and colonies counted 21 days later. Similarly, Hec50co cells untreated (*Hec50co ALDH1/PKH26(+)*, UN, *solid bar*) or pre-incubated with TEA (*Hec50co ALDH1/PKH26(+)*, PRE, *shaded bar, p ≤ 0.05*) were grown in TEA-free agar for 21 days. N = 4. (C) Untreated Ishikawa H cells with high PKH26/ALDH1 levels were seeded onto the unaltered soft agar (*Ishikawa H, ALDH1/PKH26(+)*, UN, *solid bar*) or on agar supplemented with TEA (*Ishikawa H, , ALDH1/PKH26(+)*, TEA, *shaded bar, p ≤ 0.05*) and inspected 21 days later. Similarly, PKH26/ALDH1-positive Hec50co cells on untreated agar (*Hec50co, ALDH1/PKH26(+)*, UN, *solid bar*) or agar supplemented with TEA (*Hec50co, ALDH1/PKH26(+)*, TEA, *no bar*) were counted 21 days later. N = 3.

The data in Figure 1B and 1C indicate that assaying for simultaneous high ALDH1 activity and high retention of PKH26 dye provides a better phenotypic marker for TIC than isolating cells using either marker alone. Therefore, we used this strategy to isolate TIC-enriched subpopulation and examined the effects of TEA in colony formation of double (PKH26/ALDH1) positive Ishikawa H and Hec50co cells. First, we treated cells with 10 μmol/L of TEA for 48 h, and then isolated the double positive cells via fluorescent cell sorting. As demonstrated in Figure 2A, we were able to obtain a population of cells that were positive for both PKH26 and ALDH1. The gated cells were used for subsequent experiments.

We further extended our hypothesis to examine how sustained exposure to TEA affects formation of new colonies/tumors. Due to the neurotoxicity of TEA, we could not utilize mouse xenograft models to test this hypothesis. We therefore examined growth of cell colonies in soft agar supplemented with 10 μmol/L of TEA instead. As we expected, double positive Ishikawa H and Hec50co cells developed colonies in TEA-free agar (Figure 2C, filled bars). As compared to the untreated counterparts, fewer Ishikawa H colonies grew in TEA-supplemented agar (Figure 2C, Ishikawa H, shaded bar), thus demonstrating that continuous exposure to TEA attenuated the tumorigenic ability of these cells. Remarkably, TEA completely abolished growth of Hec50co colonies (Figure 2C, Hec50co). We thus concluded that TEA abrogated the tumorigenic capacity of Ishikawa H and Hec50co cells, and the TEA withdrawal observed in Figure 1A results in augmentation of tumorigenicity.

These findings are significant for several reasons. First, they validate K^+ ^channels as candidate anti-cancer targets to maintain quiescence of TIC. In particular, cancer patients in remission that have high risk of recurrence may benefit from such therapy to prolong disease-free survival. Importantly, other inhibitors that, similar to TEA, non-selectively antagonize K^+ ^channels are FDA-approved to treat human conditions not related to cancer. These drugs may possess therapeutic benefits of TEA without the associated neurotoxicity [37]. Second, many clinically approved anti-cancer drugs may have off-target TEA-like inhibitory actions towards the variety of K^+ ^channels. Termination of therapy with these drugs may lay the foundation for cancer recurrence by virtue of enhancing the tumorigenicity of surviving TIC [37]. Intriguingly, these responses are analogous in ER-, PR-positive Ishikawa H cells and ER-, PR-negative Hec50co cells, implying a common response in molecularly disparate endometrial cancers [30]. The mechanisms whereby TEA regulates TIC function require further research, but may involve inhibitor-induced depolarization of plasmalemmal and/or mitochondrial membranes and subsequent changes in cell cycle [14]. Also, the contribution of two-pore leak K+ channels, including the K2P family, to TIC function remains undetermined since TEA does not inhibit these channels.

## Conclusions

In this study, we demonstrate that TEA, an inhibitor of K^+ ^channels, decreased tumorigenicity of human endometrial cancer cells. Importantly, withdrawal of TEA led to a significant potentiation of tumorigenic ability. Our data suggest that TEA alters tumorigenicity by inhibiting growth of TIC-enriched subpopulations with high activity of ALDH1 and a low basal proliferation rate.

## Materials and Methods

### Reagents

Tetraethylammonium chloride (TEA), crystal violet, laminin, Dulbecco's Modified Eagle Medium (DMEM):F12, gentamicin, PKH26 dye and Triton-X 100 were purchased from Sigma (St Louis, MO). ALDEFLUOR assays were from StemCell Technologies (Vancouver, BC, Canada). Agar, Hoechst 33258 and SYTO 16 were from Invitrogen (Carlsbad, CA), FBS and 0.25% trypsin/EDTA from Gibco-BRL (Carlsbad, CA). Low adherence tissue culture flasks were from A. Daigger&Company (Vernon Hills, IL) and glass bottom dishes were from MatTek Corp. (Ashland, MA).

### Cell lines

Ishikawa H cells were a generous gift from Dr. Erlio Gurpide (New York University). Hec50co cells were developed in our laboratory from parental Hec50 cells also kindly provided by Dr. Gurpide [30]. Cells were maintained in DMEM:F12 medium supplemented with 20% fetal bovine serum (FBS) and 50 μg/ml gentamicin. Antibiotic-free medium was used for the duration of experiments starting at least 24 h before experimentation.

### TEA incubations

Cells were seeded at 100,000 cells per well in 6-well cluster plates and incubated with 10^-5 ^mol/L of TEA for 48 h. Subsequently, cells were trypsinized and counted using Countess Automated Cell Counter (Invitrogen, Carlsbad, CA). Viability was determined by the trypan blue exclusion.

### ALDH1 activity assays

Aldehyde dehydrogenase isoform 1 (ALDH1) activity was assayed using ALDEFLUOR kit according to the manufacturer's instructions. Briefly, an ALDEFLUOR reagent was added to the cells. An aliquot of these cells was removed and. an ALDEFLUOR inhibitor was added to that to serve as a control for background fluorescence. Cells were incubated at 37°C for 40 min and separated into two sub-populations based on fluorescence intensity (highest ALDH1 activity, *ALDH1(+); *lowest ALDH1 activity, *ALDH1(-)*) using a 488 nm laser (FACS ARIA II, Becton Dickinson, Franklin Lakes, NJ).

### Xenograft models

Athymic NCr-nu/nu mice were purchased from NCI at Frederick (Frederick, MD). Mice were maintained in a sterile environment according to guidelines established by the US Department of Agriculture and the American Association for Accreditation of Laboratory Animal Care (AAALAC). Cells were assesses for viability using Hoechst 33258 dye and mice were injected in one flank with 100,000 *ALDH1(+) *cells and an equal amount of *ALDH1(-) *cells in the opposite flank. Tumor growth was monitored for 6-8 weeks. Two mice were used per cell line. All procedures were approved by the Institutional Animal Care and Use Committee at University of Iowa and complied with the standards stated in the Guide for the Care and Use of Laboratory Animals.

### PKH26 staining

Cell membrane staining with the fluorescent dye PKH26 was performed according to the manufacturer's instructions. Briefly, 10^6 ^cells were re-suspended in 2 mL of reagent buffer and incubated with 1 μL of PKH26. Subsequently, cells were repeatedly washed, seeded onto the tissue culture flasks and propagated for at least 7 days before experimentation.

### Fluorescent cell sorting of PKH26/ALDH1 cells

Cells were stained with the PKH26 dye for 7 days, assayed for ALDH1 activity using ALDEFLUOR reagent, and immediately analyzed by flow cytometry using 488 nm/561 nm lasers of FACS ARIA II. First, we obtained a PKH26(+) population of cells, which was then sorted to obtain ALDH1(+) cells. Cells with the highest levels of both PKH26 and ALDH1 were used in subsequent experiments.

### Endosphere cultures and imaging

Ishikawa H cells or Hec50co cells stained with PKH26 dye were seeded onto the low adherence tissue culture flasks in DMEM:F12 medium supplemented with 1% FBS. Floating cell spheres were examined 7-10 day later by confocal microscopy. Aliquots of endospheres were transferred onto 35 mm glass bottom dishes pre-coated with mouse laminin. Live cell nuclei were counter-stained with SYTO16 according to the manufacturer's instructions and individual spheres were imaged as stacks of 0.7-1 μm optical slices using 488 nm/561 nm lasers of a LSM 510 laser confocal microscope (Zeiss, Jena, Germany). Subsequently, optical slices were reconstructed into the three dimensional images using LSM Image Browser (Zeiss, Jena, Germany).

### Soft agar assays

Assays were performed in 6-well cluster tissue culture plates pre-coated with 0.5% basal agar in DMEM:F12. Cells were over-laid onto the basal agar at 1000 cells per well in DMEM:F12 supplemented with 0.3% agar and 20% FBS. Additionally, where indicated, 10^-5 ^mol/L TEA was incorporated into the cell suspension. The cell colonies (50 cells or more) were visualized 21 days later using crystal violet solution (crystal violet 0.005% and citric acid 0.1%) and counted.

### Statistical analysis

Data was plotted using SigmaPlot 11.0 software (Systat Software, Inc., San Jose, CA). Statistical significance was determined by one-way analysis of variance (ANOVA) and the post hoc Bonferroni's t-test. A *p *value <0.05 was considered statistically significant.

## Competing interests

The author declares that they have no competing interests.

## Authors' contributions

BMS performed experiments and assisted with preparation of the manuscript. NA-B performed *in vivo *experiments. KKL advised in study design. DRS advised in study designed and helped to draft the manuscript. VPK conceived of the study, participated in its design and coordination, conducted experiments, performed statistical analyses, and draft the manuscript. All authors read and approved the final manuscript.

## References

[B1] Stuhmer W Pardo LA K(+) channels as therapeutic targets in oncology Future Med Chem 2010 2 745 755 10.4155/fmc.10.24 21426201

[B2] Asher V Sowter H Shaw R Bali A Khan R Eag and HERG potassium channels as novel therapeutic targets in cancer World J Surg Oncol 2010 8 113 10.1186/1477-7819-8-113 21190577 PMC3022597

[B3] Ernest NJ Logsdon NJ McFerrin MB Sontheimer H Spiller SE Biophysical properties of human medulloblastoma cells J Membr Biol 2010 237 59 69 10.1007/s00232-010-9306-x 20931182

[B4] Bielanska J Hernandez-Losa J Perez-Verdaguer M Moline T Somoza R Ramon YCS Condom E Ferreres JC Felipe A Voltage-dependent potassium channels Kv1.3 and Kv1.5 in human cancer Curr Cancer Drug Targets 2009 9 904 914 10.2174/156800909790192400 20025600

[B5] Wondergem R Bartley JW Menthol increases human glioblastoma intracellular Ca2+, BK channel activity and cell migration J Biomed Sci 2009 16 90 10.1186/1423-0127-16-90 19778436 PMC2758849

[B6] Khaitan D Sankpal UT Weksler B Meister EA Romero IA Couraud PO Ningaraj NS Role of KCNMA1 gene in breast cancer invasion and metastasis to brain BMC Cancer 2009 9 258 10.1186/1471-2407-9-258 19640305 PMC2727533

[B7] Brevet M Haren N Sevestre H Merviel P Ouadid-Ahidouch H DNA methylation of K(v)1.3 potassium channel gene promoter is associated with poorly differentiated breast adenocarcinoma Cell Physiol Biochem 2009 24 25 32 10.1159/000227810 19590190

[B8] Nakamura M Kyo S Zhang B Zhang X Mizumoto Y Takakura M Maida Y Mori N Hashimoto M Ohno S Inoue M Prognostic impact of CD133 expression as a tumor-initiating cell marker in endometrial cancer Hum Pathol 2010 41 1516 1529 10.1016/j.humpath.2010.05.006 20800872

[B9] Chantome A Girault A Potier M Collin C Vaudin P Pages JC Vandier C Joulin V KCa2.3 channel-dependent hyperpolarization increases melanoma cell motility Exp Cell Res 2009 315 3620 3630 10.1016/j.yexcr.2009.07.021 19646982

[B10] Blackiston DJ McLaughlin KA Levin M Bioelectric controls of cell proliferation: ion channels, membrane voltage and the cell cycle Cell Cycle 2009 8 3519 3528 19823012 10.4161/cc.8.21.9888 PMC2862582

[B11] Sciaccaluga M Fioretti B Catacuzzeno L Pagani F Bertollini C Rosito M Catalano M D'Alessandro G Santoro A Cantore G Ragozzino D Castigli E Franciolini F Limatola C CXCL12-induced glioblastoma cell migration requires intermediate conductance Ca2+-activated K+ channel activity Am J Physiol Cell Physiol 2010 299 C175 184 10.1152/ajpcell.00344.2009 20392929

[B12] Becchetti A Arcangeli A Integrins and ion channels in cell migration: implications for neuronal development, wound healing and metastatic spread Adv Exp Med Biol 2010 674 107 123 10.1007/978-1-4419-6066-5_10 20549944

[B13] Haren N Khorsi H Faouzi M Ahidouch A Sevestre H Ouadid-Ahidouch H Intermediate conductance Ca2+ activated K+ channels are expressed and functional in breast adenocarcinomas: correlation with tumour grade and metastasis status Histol Histopathol 2010 25 1247 1255 20712009 10.14670/HH-25.1247

[B14] Lee I Park C Kang WK Knockdown of inwardly rectifying potassium channel Kir2.2 suppresses tumorigenesis by inducing reactive oxygen species-mediated cellular senescence Mol Cancer Ther 2010 9 2951 2959 10.1158/1535-7163.MCT-10-0511 20841375

[B15] Fortunato P Pillozzi S Tamburini A Pollazzi L Franchi A La Torre A Arcangeli A Irresponsiveness of two retinoblastoma cases to conservative therapy correlates with up- regulation of hERG1 channels and of the VEGF-A pathway BMC Cancer 2010 10 504 10.1186/1471-2407-10-504 20860824 PMC2955607

[B16] Tajima N Itokazu Y Korpi ER Somerharju P Kakela R Activity of BK(Ca) channel is modulated by membrane cholesterol content and association with Na+/K+-ATPase in human melanoma IGR39 cells J Biol Chem 2011 286 5624 5638 10.1074/jbc.M110.149898 21135099 PMC3037676

[B17] Asher V Khan R Warren A Shaw R Schalkwyk GV Bali A Sowter HM The Eag potassium channel as a new prognostic marker in ovarian cancer Diagn Pathol 2010 5 78 10.1186/1746-1596-5-78 21138547 PMC3016344

[B18] Jelassi B Chantome A Alcaraz-Perez F Baroja-Mazo A Cayuela ML Pelegrin P Surprenant A Roger S P2X(7) receptor activation enhances SK3 channels- and cystein cathepsin-dependent cancer cells invasiveness Oncogene 2011 30 2108 2122 10.1038/onc.2010.593 21242969

[B19] Lallet-Daher H Roudbaraki M Bavencoffe A Mariot P Gackiere F Bidaux G Urbain R Gosset P Delcourt P Fleurisse L Slomianny C Dewailly E Mauroy B Bonnal JL Skryma R Prevarskaya N Intermediate-conductance Ca2+-activated K+ channels (IKCa1) regulate human prostate cancer cell proliferation through a close control of calcium entry Oncogene 2009 28 1792 1806 10.1038/onc.2009.25 19270724

[B20] Asher V Warren A Shaw R Sowter H Bali A Khan R The role of Eag and HERG channels in cell proliferation and apoptotic cell death in SK-OV-3 ovarian cancer cell line Cancer Cell Int 2011 11 6 10.1186/1475-2867-11-6 21392380 PMC3063814

[B21] Kim HJ Jang SH Jeong YA Ryu PD Kim DY Lee SY Involvement of Kv4.1 K(+) channels in gastric cancer cell proliferation Biol Pharm Bull 2010 33 1754 1757 10.1248/bpb.33.1754 20930388

[B22] Biderman B Marakhonov A Skoblov M Birerdinc A Nohelty E Page S Khomenkov V Chandhoke V Sudarikov A Nikitin E Baranova A Inhibition of potassium currents as a pharmacologic target for investigation in chronic lymphocytic leukemia Drug News Perspect 2010 23 625 631 10.1358/dnp.2010.23.10.1507740 21180648

[B23] Pillozzi S Masselli M De Lorenzo E Accordi B Cilia E Crociani O Amedei A Veltroni M D'Amico M Basso G Becchetti A Campana D Arcangeli A Chemotherapy resistance in acute lymphoblastic leukemia requires hERG1 channels and is overcome by hERG1 blockers Blood 2011 117 902 914 10.1182/blood-2010-01-262691 21048156

[B24] Agarwal JR Griesinger F Stuhmer W Pardo LA The potassium channel Ether a go-go is a novel prognostic factor with functional relevance in acute myeloid leukemia Mol Cancer 2010 9 18 10.1186/1476-4598-9-18 20105281 PMC2835655

[B25] Jang SH Kang KS Ryu PD Lee SY Kv1.3 voltage-gated K(+) channel subunit as a potential diagnostic marker and therapeutic target for breast cancer BMB Rep 2009 42 535 539 10.5483/BMBRep.2009.42.8.535 19712592

[B26] Dolderer JH Schuldes H Bockhorn H Altmannsberger M Lambers C von Zabern D Jonas D Schwegler H Linke R Schroder UH HERG1 gene expression as a specific tumor marker in colorectal tissues Eur J Surg Oncol 2010 36 72 77 19577877 10.1016/j.ejso.2009.05.009

[B27] Teng IW Hou PC Lee KD Chu PY Yeh KT Jin VX Tseng MJ Tsai SJ Chang YSD Wu CS Sun HS Tsai KD Jeng LB Nephew KP Huang TH Hsiao SH Leu YW Targeted methylation of two tumor suppressor genes is sufficient to transform mesenchymal stem cells into cancer stem/initiating cells Cancer Res 2011 10.1158/0008-5472.CAN-10-3418 21518779

[B28] Kano M Tsukahara T Emori M Murase M Torigoe T Kawaguchi S Wada T Yamashita T Sato N Autologous CTL response against cancer stem-like cells/cancer-initiating cells of bone malignant fibrous histiocytoma Cancer Sci 2011 10.1111/j.1349-7006.2011.01962.x 21518139

[B29] Deleyrolle LP Harding A Cato K Siebzehnrubl FA Rahman M Azari H Olson S Gabrielli B Osborne G Vescovi A Reynolds BA Evidence for label-retaining tumour-initiating cells in human glioblastoma Brain 2011 134 1331 1343 10.1093/brain/awr081 21515906 PMC3097894

[B30] Albitar L Pickett G Morgan M Davies S Leslie KK Models representing type I and type II human endometrial cancers: Ishikawa H and Hec50co cells Gynecol Oncol 2007 106 52 64 10.1016/j.ygyno.2007.02.033 17490735

[B31] Cherubini A Taddei GL Crociani O Paglierani M Buccoliero AM Fontana L Noci I Borri P Borrani E Giachi M Becchetti A Rosati B Wanke E Olivotto M Arcangeli A HERG potassium channels are more frequently expressed in human endometrial cancer as compared to non-cancerous endometrium Br J Cancer 2000 83 1722 1729 10.1054/bjoc.2000.1497 11104572 PMC2363441

[B32] Wang ZH Shen B Yao HL Jia YC Ren J Feng YJ Wang YZ Blockage of intermediate-conductance-Ca(2+) -activated K(+) channels inhibits progression of human endometrial cancer Oncogene 2007 26 5107 5114 10.1038/sj.onc.1210308 17310992

[B33] Pece S Tosoni D Confalonieri S Mazzarol G Vecchi M Ronzoni S Bernard L Viale G Pelicci PG Di Fiore PP Biological and molecular heterogeneity of breast cancers correlates with their cancer stem cell content Cell 2010 140 62 73 10.1016/j.cell.2009.12.007 20074520

[B34] Heerma van Voss MR van der Groep P Bart J van der Wall E van Diest PJ Expression of the stem cell marker ALDH1 in BRCA1 related breast cancer Cell Oncol (Dordr) 2011 34 3 10 10.1007/s13402-010-0007-3 PMC3046359 21336637

[B35] Rahadiani N Ikeda J Mamat S Matsuzaki S Ueda Y Umehara R Tian T Wang Y Enomoto T Kimura T Aozasa K Morii E Expression of aldehyde dehydrogenase 1 (ALDH1) in endometrioid adenocarcinoma and its clinical implications Cancer Sci 2011 102 903 908 10.1111/j.1349-7006.2011.01864.x 21231983

[B36] Kusumbe AP Bapat SA Cancer stem cells and aneuploid populations within developing tumors are the major determinants of tumor dormancy Cancer Res 2009 69 9245 9253 10.1158/0008-5472.CAN-09-2802 19951996

[B37] Potier M Chantome A Joulin V Girault A Roger S Besson P Jourdan ML LeGuennec JY Bougnoux P Vandier C The SK3/K(Ca)2.3 potassium channel is a new cellular target for edelfosine Br J Pharmacol 2011 162 464 479 10.1111/j.1476-5381.2010.01044.x 20955368 PMC3031066

